# Supplementary material for: ReadZS detects cell type-specific and developmentally regulated RNA processing programs in single-cell RNA-seq
Source: Genome Biol. 2022 Oct 25;23:226. doi: 10.1186/s13059-022-02795-8 (PMC9594907; doi:10.1186/s13059-022-02795-8)
Supplement: Supplementary file 1 — Additional file 1: Supplementary figures. This file contains all supplementary figures, as well as in-depth descriptions of the supplementary tables (Additional files 2, 3, 4 and 5). [file 13059_2022_2795_MOESM1_ESM.pdf]

Figure S1

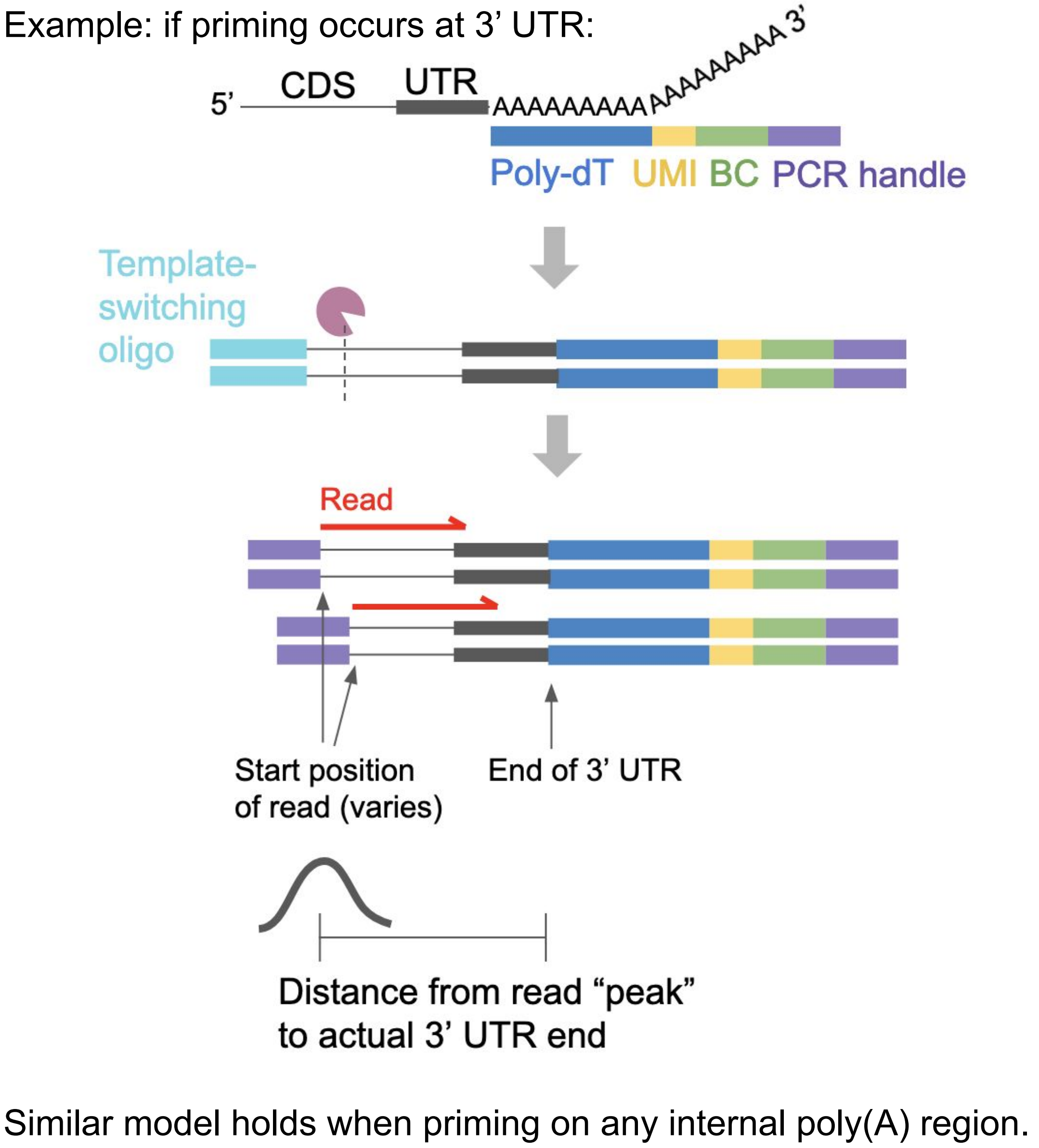

Figure S1: Preparation of 10X sequencing libraries results in varying distance between start of read and actual end of 3' UTR. Example, if priming at the end of a 3' UTR (top to bottom): RNA transcript pairs to the poly(dT) sequence (shown in blue); the first arrow represents multiple steps including reverse transcription, template switching, and cDNA amplification; the resulting cDNA is enzymatically cleaved at different sites, resulting in a range of start positions for the final reads (represented in red). As a result, the distance from the start position of a read and the actual 3' end of the 3' UTR will vary, but can be predicted based on the final length range of the inserts and the sequencing read length. Note that priming can occur at any poly(A) region including internally.

Figure S2

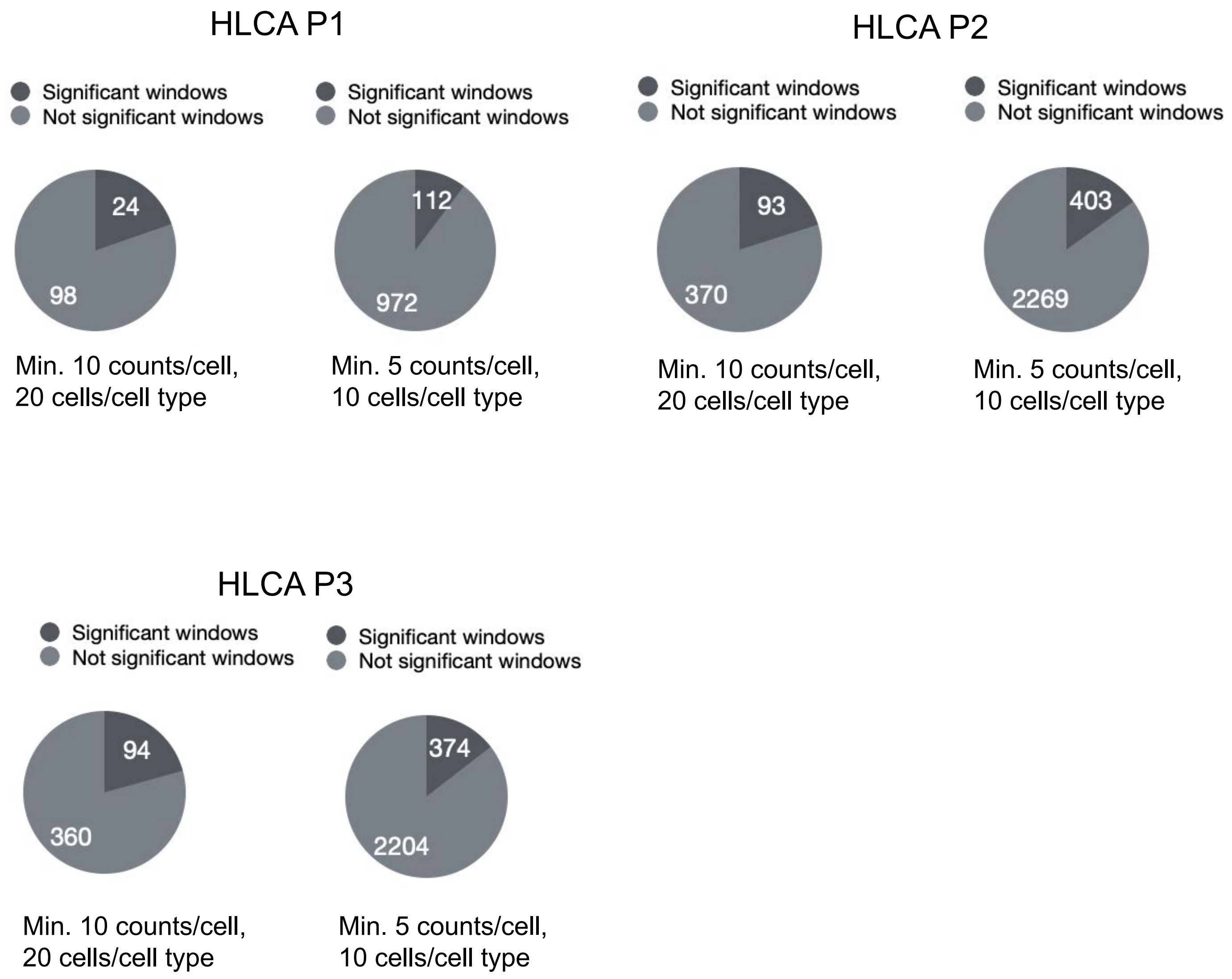

Figure S2: Summary: proportion of genomic windows that were called by ReadZS as having significant cell type-specific RNAP, in HLCA P1, P2, and P3, with either the standard minimum 10 counts per cell and 20 cells per cell type, or with the reduced minimum of 5 counts per cell and 10 cells per cell type.

Figure S3

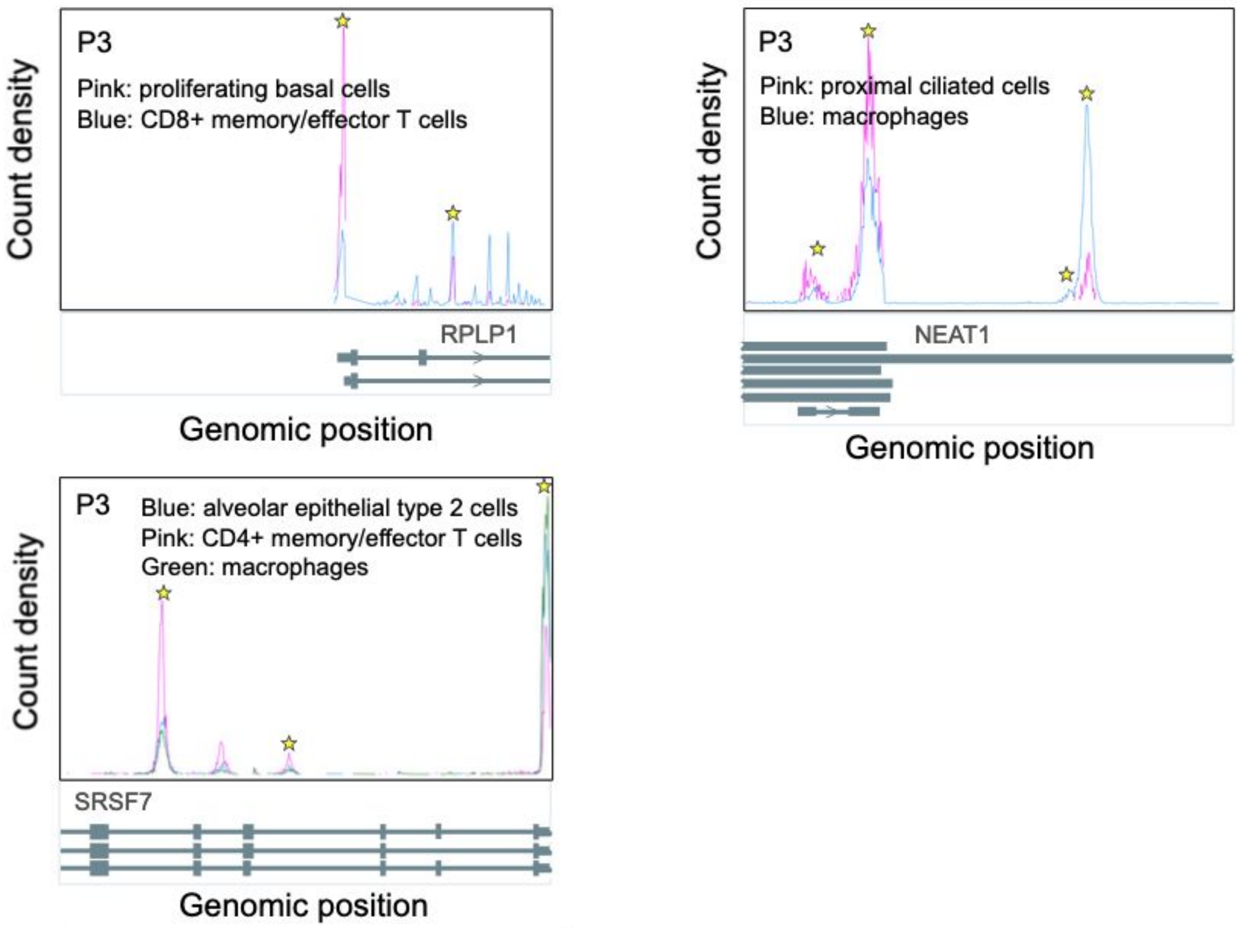

Figure S3: Read distributions of genomic windows in P3 with significantly different cell type-specific RNA processing, overlapping the genes *RPLP1*, *NEAT1*, and *SRSF7* (left to right). Peaks in significant windows called by the GMM are starred; peak calls are done using the combined data from all cells across all cell types (Methods). Because P3 and P2 have different distributions of cell types and read depths within those cell types, these windows did not have sufficient reads in the same cell types in P2 to compute median ReadZS values.

Figure S4

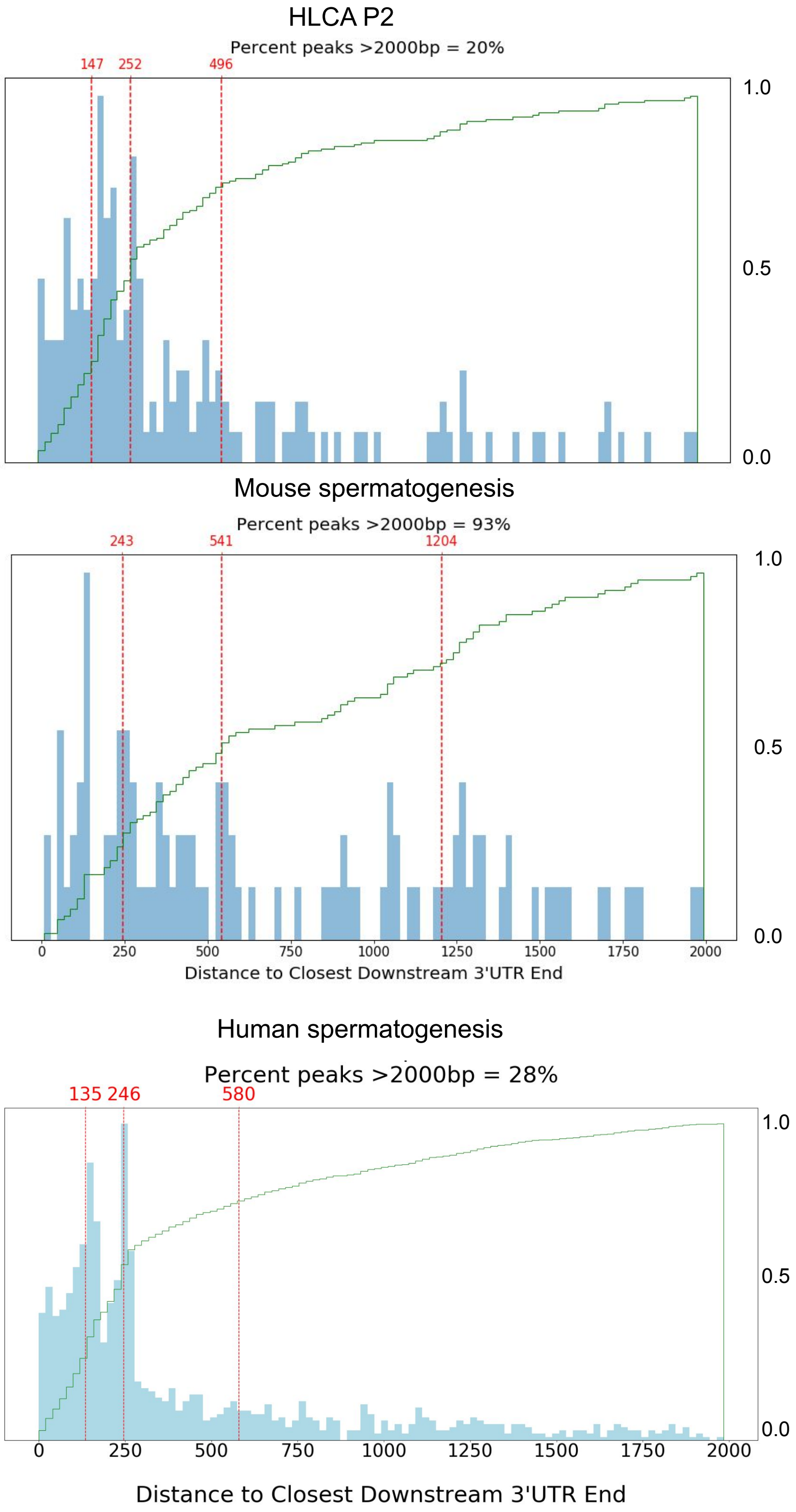

Figure S4: Histogram and cdfs of the distribution of distances from GMM-called peaks to closest downstream annotated 3' UTR in HLCA P2, mouse spermatogenesis, and human spermatogenesis; lines denote the 25th, 50th, and 75th quantile, respectively. Distance distributions are compatible with expectation from 10x library construction.

Figure S5

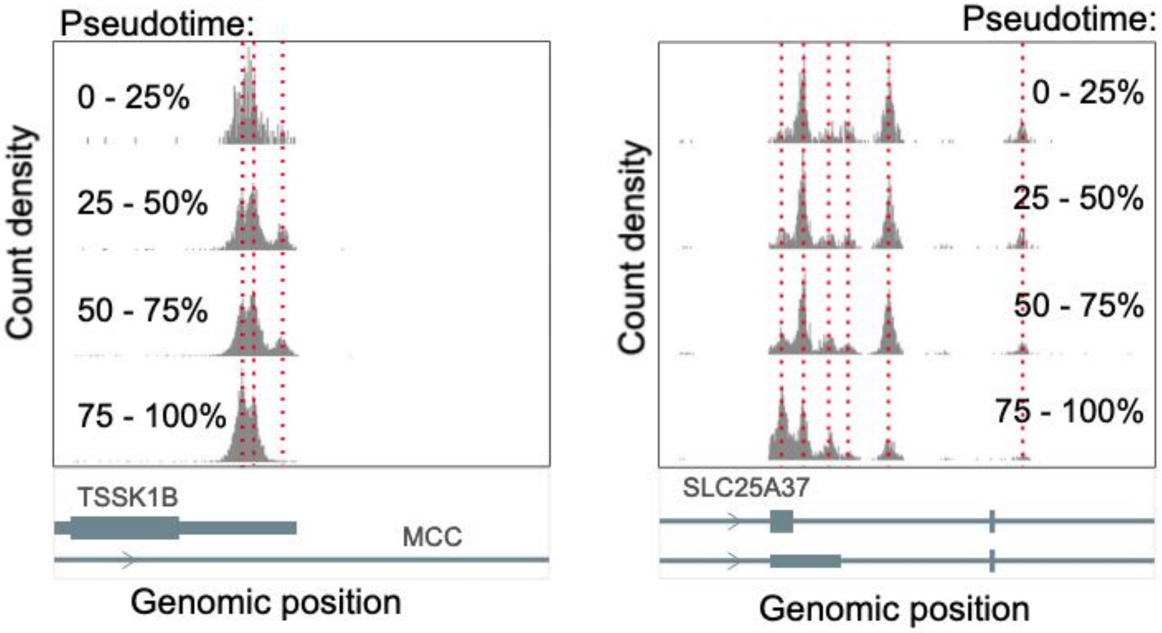

Figure S5: Read distributions of genomic windows in human spermatogenesis with significant correlation between ReadZS and pseudotime. Left: window overlapping *TSSK1B* (Spearman's correlation = -0.549, Bonferroni-corrected p-value < 1E-82); right: window overlapping *SLC25A37* (Spearman's correlation = -0.512, Bonferroni-corrected p-value < 1E-35). Red lines are placed to highlight peaks in read distribution.

Figure S6

(A)

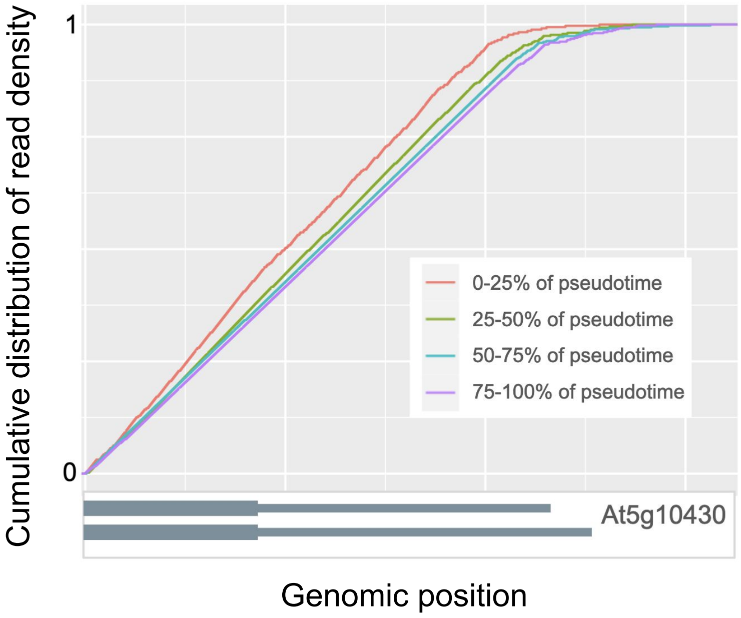

(B)

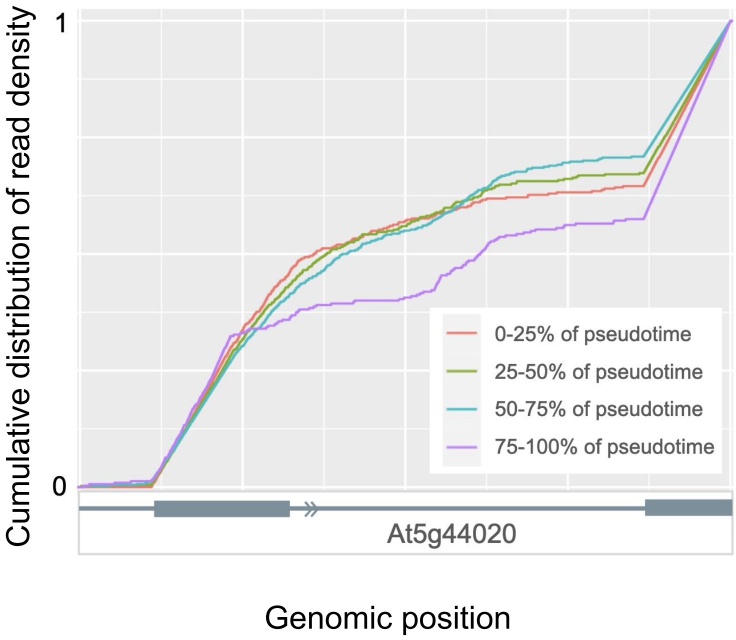

Figure S6: (A) Cumulative distribution functions of read positions from a genomic window in Arabidopsis root with significant correlation between ReadZS and pseudotime: in atrichoblasts, genomic window overlapping the gene *At5g10430* (Spearman's correlation = 0.658, Bonferroni-corrected p-value < 0.0001), from library sc_9_at. (B) Cumulative distribution functions of read positions from a genomic window in Arabidopsis root with significant correlation between ReadZS and pseudotime: in trichoblasts, genomic window overlapping the gene *At5g44020* (Spearman's correlation = 0.620 Bonferroni-corrected p-value < 0.0001), from library sc_11.

Figure S7

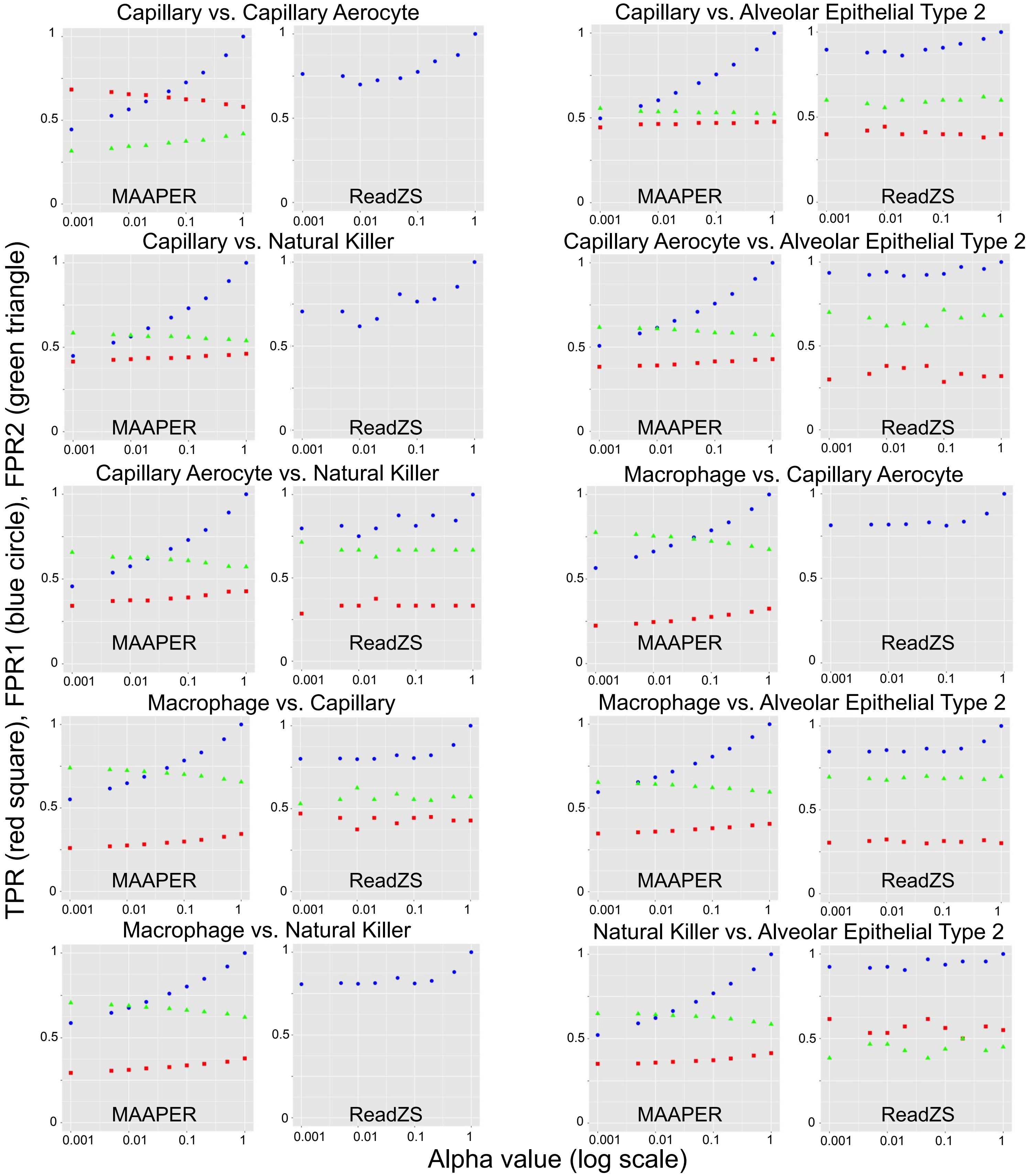

Figure S7: Proxy measurements for true and false positive rates of MAAPER and ReadZS, from comparisons of different pairs of cell types from HLCA P2 and P3, evaluated at different alpha values (corrected p-value cutoffs for significance): "true positive rate" proxy (TPR) - red squares; "false positive rate" proxy 1 (FPR1) - blue circles; and "false positive rate" proxy 2 (FPR2) - green triangles (see Methods for calculation of these metrics). Missing points indicate that there were not sufficient significant genes or windows to calculate the proxy measurement.

Figure S8

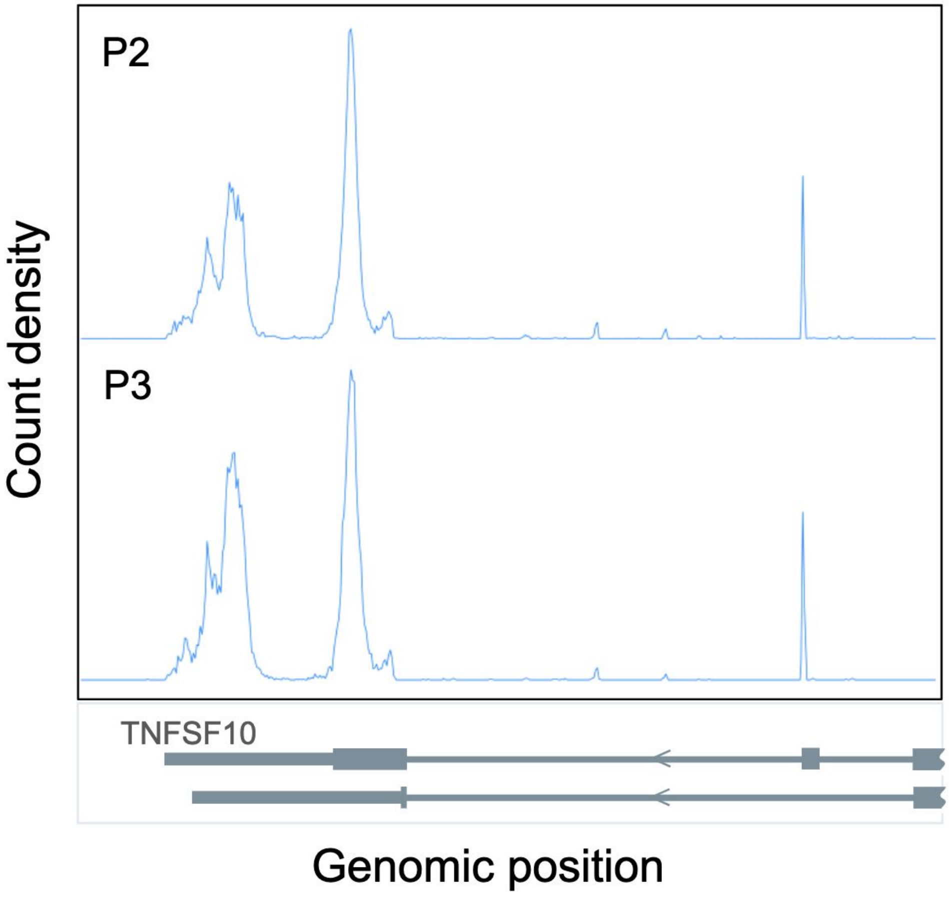

Figure S8: When comparing capillary cells in HLCA P2 vs. HLCA P3, this genomic window overlapping the end of gene *TNFSF10* was called as significant by ReadZS. Even though this significant window would count toward the proxy “FPR”, there is clearly a difference in read distribution corresponding to expression of different isoforms of this gene.

Figure S9

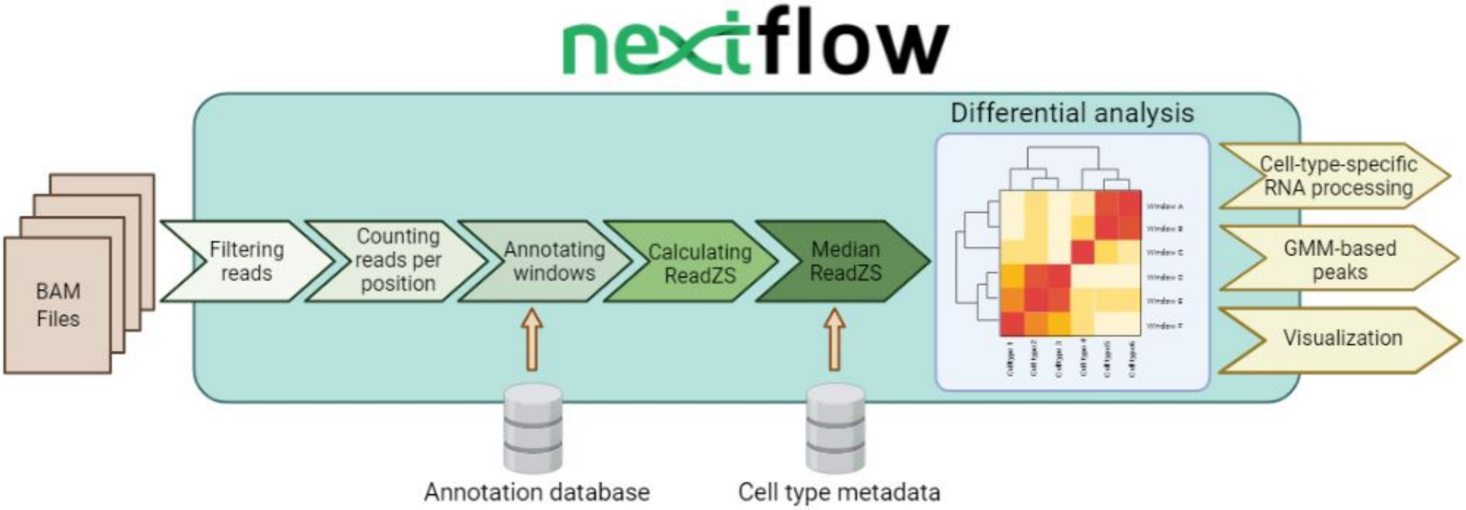

Figure S9: The Nextflow-based implementation of ReadZS allows for scalable, portable, and reproducible identification of cell type-specific RNA processing events without the need for pre-installation of software and packages.

Supplementary table descriptions:

<u>Table S1:</u> cell type-specific RNA processing events in HLCA dataset.

- Sheet 1 ("P3 significant windows"): list of windows in HLCA P3 with significant cell type-specific RNAP. There is one for each pair of window and cell type. The columns are as follows:
  chr_window: chromosome and bin number of the genomic window
  window: name of genomic window (composed of chromosome, end position, and strand)
  ontology: cell type as defined by metadata
  median_ReadZS: median ReadZS for this window by ontology
  gene: gene overlapping this window, based on intersecting with RefSeq annotations
- Sheet 2 ("P3 peaks in signif windows"): list of peaks called in significant windows from HLCA P3. There is one line per called peak from each significant window. All intersections and nearest annotations are strand-specific to the window. The columns are as follows:
  window: name of genomic window (composed of chromosome, end position, and strand)
  start: start position of the genomic window
  end: end position of the genomic window
  num_peaks: number of GMM-called peaks
  peak_pos: GMM-called peak position
  num_3UTR_300bp_downstream: number of downstream 3'UTR ends intersecting 300bp downstream of the genomic window
  window_has_gene: TRUE if the genomic window intersects with an annotated gene
  peak_has_600bp_downstream_gene: TRUE any region 600bp downstream of the GMM-called peak intersects with an annotated gene
  upstream_3UTR: nearest upstream annotated 3'UTR end to the called peak
  upstream_3UTR_dist: distance to the nearest upstream annotated 3'UTR end to the called peak
  downstream_3UTR: nearest downstream annotated 3'UTR end to the called peak
  downstream_3UTR_dist: distance to the nearest downstream annotated 3'UTR end to the called peak
  ICL_vec: the vector of ICL criterion values for each number if components
- Sheet 3 ("P2 significant windows"): list of windows in HLCA P2 with significant cell type-specific RNAP. There is one line per median, so there are multiple lines for each window. This table includes only significant windows. The columns are the same as Sheet 1.
- Sheet 4 ("P2 peaks in signif windows"): list of peaks called by GMM in significant windows from HLCA P2. There is one line per called peak from each significant window. All intersections and nearest annotations are strand-specific to the window. The columns are the same as Sheet 2.

<u>Table S2:</u> Regulated RNA processing in human and mouse spermatogenesis.

- Sheet 1 ("human - significant windows"): The correlation of windows with pseudotime in human spermatogenesis data. This table includes both significant and not significant windows. The correlation coefficients are not adjusted for window direction here. The columns are as follows:
  window: name of genomic window (composed of chromosome, end position, and strand)
  median_counts_per_cell: median number of reads in this window per cell
  spearman: Spearman's correlation between ReadZS and pseudotime for this window

pearson: Pearson correlation between ReadZS and pseudotime for this window
spearman_pvalue: Bonferroni-adjusted p-value for Spearman's correlation
pearson_pvalue: Bonferroni-adjusted p-value for pearson correlation

- Sheet 2 ("human - peaks in signif windows"): list of peaks called by GMM in significantly correlated windows from human spermatogenesis data. There is one line per called peak from each significant window. All intersections and nearest annotations are strand-specific to the window and the columns are the same as those in Tables 1 and 2.
- Sheets 3 ("mouse - significant windows") and 4 ("mouse - peaks in signif windows"): similar tables as Sheets 1 and 2 but for mouse spermatogenesis data.

<u>Table S3:</u> Regulated RNA processing in Arabidopsis root development.

- Sheet 1 ("sc_9_at window correlations"): The correlation of windows with pseudotime in library sc_9_at from the Arabidopsis root data. This table includes both significant and not significant windows. The correlation coefficients are not adjusted for window direction here. The columns are as follows:
  window: name of genomic window (composed of chromosome, end position, and strand)
  celltype: Arabidopsis root cell type
  median_counts_per_cell: median number of reads in this window per cell
  spearman: Spearman's correlation between ReadZS and pseudotime for this window
  pearson: Pearson correlation between ReadZS and pseudotime for this window
  spearman_pvalue: Bonferroni-adjusted p-value for Spearman's correlation
- Sheet 2 ("sc_10_at window correlations"): The correlation of windows with pseudotime in library sc_10_at. Same columns as Sheet 1.
- Sheet 3 ("sc 1,9,10,11 window corr"): The correlation of windows with pseudotime in libraries sc_1, sc_9_at, sc_10_at, and sc_11. Same columns as Sheet 1.
- Sheet 4 ("sc 1,9,10,11 gene corr"): The correlation of *genes* with pseudotime in libraries sc_1, sc_9_at, sc_10_at, and sc_11. The correlations in this table are calculated from ReadZS values from using ReadZS based on genes instead of evenly spaced genomic windows. Same columns as Sheet 1 except that "window" is replaced by "gene_window", where each gene is recorded in the format [chr]_[gene name]_[strand].

<u>Table S4:</u> cell type-specific RNA processing events in mouse fibroblast dataset.

- Sheet 1 ("mouse fibr. - significant windows"): list of windows in mouse fibroblast data found to have significant cell type-specific RNAP. Columns are the same as Sheet 1 in Table 1.
- Sheet 2 ("mouse fibr. - medians with ann"): table of median ReadZS values for each pair of window and cell type There is one line per median, so there are multiple lines for each window. This table includes both significant and not significant windows. The columns are as follows:
  window = window name
  ontology = cell type
  median_ReadZS = median ReadZS value
  chi2_p_val = p-value by chi^2 test
  perm_p_val = p-value by permutation; might be NA if chi^2 p-value wasn't low enough
  significant = whether or not this window was called as significant
  gene = name of gene intersecting this window, as found by intersecting with RefSeq annotations; if none, this field is filled by .
  UTR3 = name of 3'UTR intersecting this window, as found by intersecting with RefSeq; if none, this field is filled by .
  in_sierra_FAct_vs_resting = is this window in the list of genes called in the Sierra paper as having DTU between F-Act fibroblasts and resting fibroblasts

in_sierra_FCyc_vs_resting = is this window in the list of genes called in the Sierra paper as having DTU between F-Cyc fibroblasts and resting fibroblasts
in_sierra_FCI_vs_resting = is this window in the list of genes called in the Sierra paper as having DTU between F-CI fibroblasts and resting fibroblasts
in_sierra_PCR_genes = is this window in the list of genes tested by RT-qPCR in the Sierra paper

- Sheet 3 (“mouse fibr. - peaks in signif windows”): list of peaks called by GMM in significant windows from Sierra data. There is one line per called peak from each significant window. All intersections and nearest annotations are strand-specific to the window. The columns are as follows:
  window: name of genomic window (composed of chromosome, end position, and strand)
  start: start position of the genomic window
  end: end position of the genomic window
  num_peaks: number of GMM-called peaks
  peak_pos: GMM-called peak position
  num_3UTR_300bp_downstream: number of downstream 3’UTR ends intersecting 300bp downstream of the genomic window
  window_has_gene: TRUE if the genomic window intersects with an annotated gene
  peak_has_600bp_downstream_gene: TRUE any region 600bp downstream of the GMM-called peak intersects with an annotated gene
  upstream_3UTR: nearest upstream annotated 3’UTR end to the called peak
  upstream_3UTR_dist: distance to the nearest upstream annotated 3’UTR end to the called peak
  downstream_3UTR: nearest downstream annotated 3’UTR end to the called peak
  downstream_3UTR_dist: distance to the nearest downstream annotated 3’UTR end to the called peak
  ICL_vec: the vector of ICL criterion values for each number if components
